# Evolution Trend of Brain Science Research: An Integrated Bibliometric and Mapping Approach

**DOI:** 10.1002/brb3.70451

**Published:** 2025-05-21

**Authors:** Sujuan Zhang, Jingyan Gu, Yang Yang, Jiangan Li, Lulu Ni

**Affiliations:** ^1^ Institute of Science and Technology Information Beijing Academy of Science and Technology Beijing China; ^2^ Department of Basic Medicine Jiangnan University Wuxi People's Republic of China; ^3^ Department of Emergency Wuxi No. 2 People's Hospital Wuxi People's Republic of China

**Keywords:** bibliometrics, brain science, research hotspot

## Abstract

**Background:**

Brain science research is considered the crown jewel of 21st‐century scientific research; the United States, the United Kingdom, and Japan have elevated brain science research to a national strategic level. This study employs bibliometric analysis and knowledge graph visualization to map global trends, research hotspots, and collaborative networks in brain science, providing insights into the field's evolving landscape and future directions.

**Methods:**

We analyzed 13,590 articles (1990–2023) from the Web of Science Core Collection using CiteSpace and VOSviewer. Metrics included publication volume, co‐authorship networks, citation patterns, keyword co‐occurrence, and burst detection. Analytical tools such as VOSviewer, CiteSpace, and online bibliometric platforms were employed to facilitate this investigation.

**Results:**

The United States, China, and Germany dominated research output, with China's publications rising from sixth to second globally post‐2016, driven by national initiatives like the *China Brain Project*. However, China exhibited limited international collaboration compared to the United States and European Union. Key journals included *Human Brain Mapping* and *Journal of Neural Engineering*, while emergent themes centered on “task analysis,” “deep learning,” and “brain–computer interfaces” (BCIs). Research clusters revealed three focal areas: (1) *Brain Exploration* (e.g., fMRI, diffusion tensor imaging), (2) *Brain Protection* (e.g., stroke rehabilitation, amyotrophic lateral sclerosis therapies), and (3) *Brain Creation* (e.g., neuromorphic computing, BCIs integrated with AR/VR). Despite China's high output, its influence lagged in highly cited scholars, reflecting a “quantity‐over‐quality” challenge.

**Conclusion:**

Brain science research is in a golden period of development. This bibliometric analysis offers the first comprehensive review, encapsulating research trends and progress in brain science. It reveals current research frontiers and crucial directions, offering a strategic roadmap for researchers and policymakers to navigate countries when planning research layouts.

## 1 Introduction

Brain science, closely related to neuroscience, is an interdisciplinary field exploring the nervous system, especially the brain. Neuroscience leans towards biological and physiological aspects, studying neural mechanisms at cellular and molecular levels. Brain science, however, has a broader scope, integrating elements from psychology, cognitive science, and engineering to understand the brain's complex functions and interactions (Kandel et al. 2021, Crick 1994, Gazzaniga 2000). This article uses the term “brain science” to emphasize its contemporary research characteristics that are intertwined with information technology. This field is a crucial part of scientific exploration, often called the “ultimate frontier” for understanding humanity and nature (Churchland 2013). It exemplifies how traditional disciplines are re‐energized through multidisciplinary convergence, standing as a major 21st‐century scientific frontier.

Brain science research is of great significance. It helps us better understand ourselves and contributes to preventing and treating neurological and mental disorders. Research on neurodegenerative diseases like Alzheimer's and Parkinson's has advanced significantly (Tuszynski 2024, Eisenstein 2024). Also, its combination with information technology and engineering has created emerging fields, such as neuromorphic intelligence, which is vital for future intelligent societies (Vassilis 2024). Globally, brain science research has been actively promoted. In 2022, significant progress was made in brain science and neuromorphic intelligence, as seen in the work of the European Commission (2022) and the National Institutes of Health (2022). The main aim is to deepen our understanding of the brain, focusing on “knowing, protecting, and creating the brain.”

Currently, knowledge graphs are being applied in brain science research. For example, the Chinese Academy of Sciences' Institute of Automation's Neuromorphic Intelligence Research Center has implemented a knowledge graph for brain science research, and the Human Brain Project has had EBRAINS Knowledge Graph (https://kg.ebrains.eu) (Amunts et al. 2019). Their main function is to utilize the knowledge graph method to provide data integration, sharing, and search services. However, in the current stage, knowledge graphs in the field of brain science research also face the need for improvement, such as the failure to better use knowledge graphs for reasoning and mining data and the problem of insufficient maintenance of the platform.

Although many scholars have made great contributions to clarifying research in the field of brain science, the development of brain science research is advancing rapidly. What is the current trend in global brain science development? What are the future trends in brain science? However, these issues need to be addressed. In the past, most literature reviews in the field of brain science research focused on qualitative analysis, which had certain limitations in terms of subjective evaluation. Recently, the emerging knowledge graph method has provided new methods and ideas to address these shortcomings and has been widely applied in various disciplines and fields. In view of this, this article systematically sorts out the relevant literature in the field of brain science research and uses the knowledge graph method to present the overall pattern and hotspots of brain science research in a visual way to provide a theoretical reference for future scholars' related research.

## 2 Data Sources and Analysis Tools

### 2.1 Data Sources

By querying Chinese databases such as CNKI, it can be found that the total number of research papers in Chinese literature with “brain science research” as the main topic is relatively small and scattered. The research frontiers of domestic scholars and research institutions are mostly published in journals indexed by the Science Citation Index (SCI) and the Social Sciences Citation Index (SSCI) abroad. Therefore, this study focuses on collecting data from the SCI database of the ISI Web of Science in the United States, which includes nearly 6,000 core academic journals in 170 fields worldwide. This article selects English literature data sources from the Science Citation Index Expanded (SCI‐EXPANDED) and SSCI, two sub‐databases in the authoritative database Web of Science Core Collection. Data collection and analysis were carried out in three steps: searching for and defining data, extracting and cleaning data, and analyzing data.

The specific search strategy is as follows: first, enter Web of Science and set the time limit for the period of the journals in the search control conditions from January 1, 1990 to February 10, 2023. Second, set the subject term to “brain science” and select “article” and “review” as document types for a combined search. Based on these conditions, the initial screening yielded 13,780 relevant articles. By removing duplicates and incomplete or irrelevant articles through machine learning and manual review, 13,590 articles were obtained after repeated refinement of the search, with the search date as of February 10, 2023. The data were loaded into the Web of Science “download_1‐13590” format and separated into different date “input–output” information units. This resulted in the identification of entity layers required for building a node network measurement model, including researchers, research institutions, geographic regions, and carriers. In addition, theme layers were identified, which included elemental units such as hot topics, keywords, themes, and research fields.

### 2.2 Analysis Tools

**FIGURE 1:**
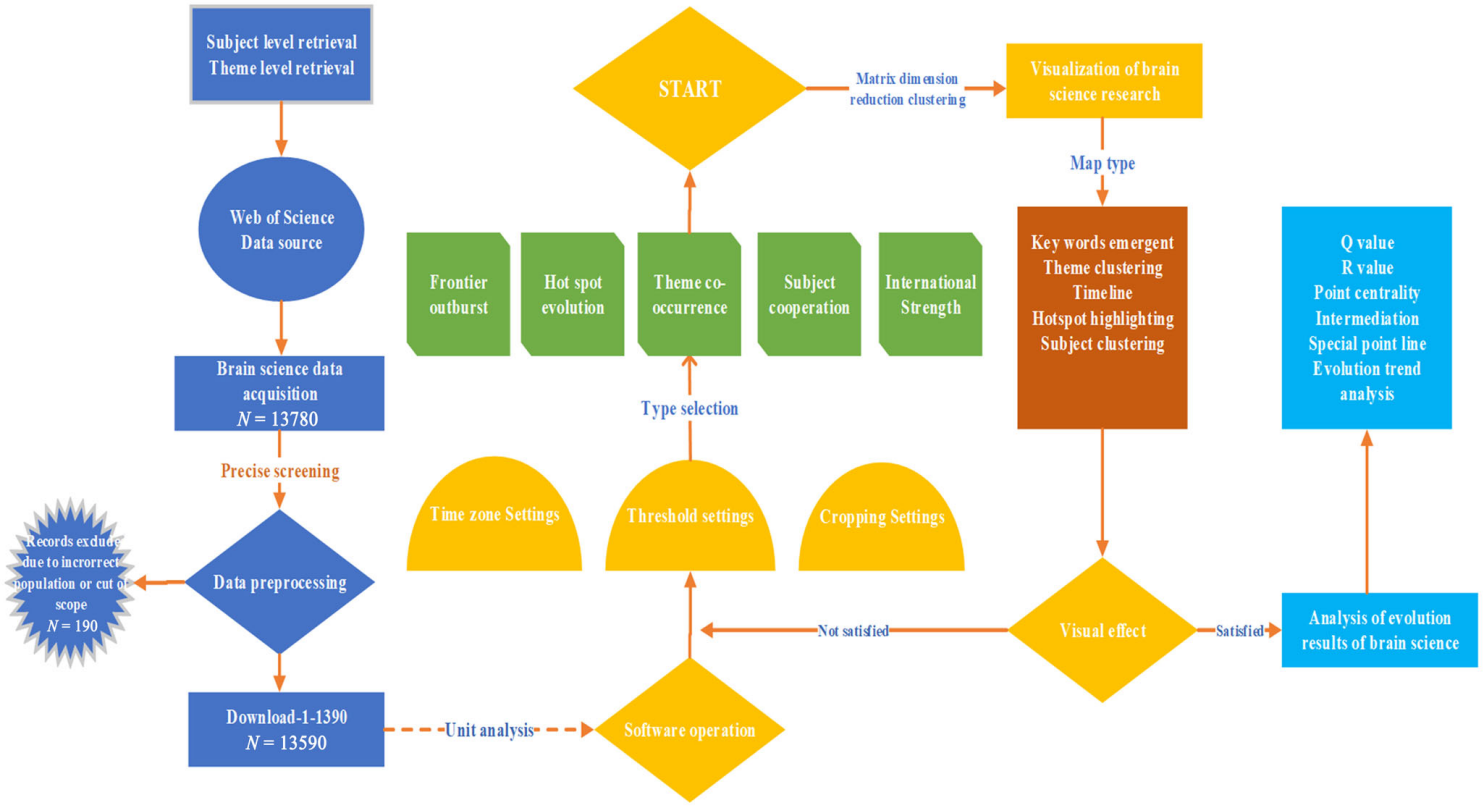
The visualization operation process of the derivation diagram of brain science research. Data collection and analysis are carried out in three steps: searching and defining data, extracting and cleaning data, and analyzing data. Type selection includes frontier outburst, hotspot evolution, theme co‐occurrence, subject cooperation, international strength, and so on. Visual topic types include keywords emergent, theme clustering, timeline, hot spot highlighting, subject clustering, and so on.

The knowledge graph method combines applied mathematics, graphics, information visualization techniques, and bibliometric analysis to intuitively display the inherent logical relationships between research topics in a certain field in the form of a multidimensional structured graph to understand the evolutionary path, research hotspots, and future trends of the field. This study used Cite Space, a citation analysis tool jointly developed by the DUT‐Drexel Joint Institute for the Study of Knowledge Visualization and Scientific Discovery in the United States, and VOSviewer, a tool developed by the Center for Science and Technology Studies at the University of Leiden in the Netherlands, to achieve one‐mode undirected network analysis of the subject and topic layers. The two software programs were developed based on methodologies such as knowledge mapping structure and psychological proximity rules. Through the representation of unit pattern mutual neighbor relationships, relationship strength, and density tests, multidimensional spatial concept information sources from brain science research are transformed into a two‐dimensional plane visualization, which is conducive to highlighting the current core points of brain science research and clarifying the research status of the neuroscientific knowledge domain (Figure 1). At present, the two software programs have become mainstream for drawing knowledge maps, with many advantages such as convenient use and high scientific efficiency. They can visualize the hotspots and frontiers of research areas, which meets the research needs of this article (He et al. 2022, X.‐L. Li et al. 2022, Y. Zhang, Chen, Tian, et al. 2022).

## 3 Results and Analysis

### 3.1 The Hierarchical Structure of the Main Subjects in Brain Science Research

#### 3.1.1 Stages of Development of Brain Science Research

**FIGURE 2:**
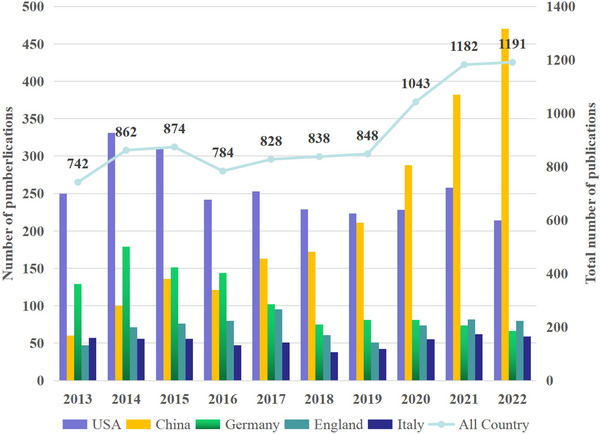
The annual publications indexed in the WOS from 2013 to 2022 and the article number of the top 5 countries/regions with “brain sciences” are presented.

The number of publications can intuitively measure the developmental trend of the brain science field over the past decade. The peaks and inflection points of the curve can reflect the changes in the research hotspots of the field, which is of great significance for analyzing and predicting the development trend of the brain science field. From the first literature on the topic of brain science research from 1991 until the end of 2012, a period of 22 years, the leading countries in the field of brain science research were the United States, Germany, England, Italy, and France (Table 1). China ranked sixth globally, with 259 papers. However, in the past decade, from 2013 to the end of 2022, the top five countries in terms of publication volume, from high to low, were the United States (2540 papers), China (2103 papers), Germany (1082 papers), the United Kingdom (717 papers), and Canada (528 papers). China's ranking has risen by four, and its total publication volume ranks second. Canada and Australia slightly improved their rankings, while Austria's ranking dropped significantly. The United States, China, Germany, the United Kingdom, and Canada formed the first camp in brain science research in the past decade. Furthermore, China ranked third in brain science research publication volume among the five countries in 2013, with a particularly significant gap with the United States (Figure 2). However, since 2017, China's publication volume has continuously surpassed that of Germany, the United Kingdom, and Italy, and the gap between China and the United States has gradually narrowed. Especially during the COVID‐19 pandemic from 2020 to 2022, when the publication volumes of the United States, Germany, the United Kingdom, Italy, and other countries decreased or stagnated to varying degrees, China's publication volume continued to grow rapidly. Since 2020, China has taken the lead and has gradually widened its gap with the second‐ranked United States. China has seen the fastest rise in brain science research publication volume in the past decade, especially in the past 5 years, with the most outstanding performance. To explain the reason, thanks to the release of “The 13th Five‐Year Plan” by the Chinese government in March 2016, which listed “brain science and brain‐like research” as a “major national science and technology innovation and engineering project,” marking the full launch of the “China Brain Project.”

**TABLE 1:** The world's major countries in two rounds of publication ranking and changes in the brain sciences.

Country	Total publications from 1991 to 2012	Ranking from 1991 to 2012	Total publications from 2013 to 2022	Ranking from 2013 to 2022	Change in rank
USA	1781	1	2540	1	0
Germany	730	2	1082	3	−1
England	341	3	717	4	−1
Italy	282	4	523	6	−2
France	275	5	489	7	−2
China	259	6	2103	2	4
Japan	257	7	472	9	−2
Canada	243	8	528	5	3
The Netherlands	195	9	352	11	−2
Austria	188	10	209	14	−4
Switzerland	135	11	290	13	−2
Spain	116	12	375	10	2
South Korea	99	13	483	8	5
Belgium	85	14	182	15	−1
Australia	83	15	315	12	3

#### 3.1.2 “Low Cohesion, High Separation” in Brain Science Researcher Networks

Co‐authorship analysis is helpful in clarifying the current composition of research teams and indicates the maturity of the brain science field, which is a multidimensional reflection of researchers' contributions. Using VOSviewer to create a co‐authorship model, a balloon heat map was selected to represent different research teams in different colors, with grayscale indicating continuous exploration of brain science research or smaller amounts of research text. The larger the value of the ball, the higher the researcher's publication volume and contribution to the field. The arcs are used to indicate cooperation between individual nodes, with the thickness of the arcs reflecting the degree of collaboration between the nodes, thus showing the construction and development of a collaborative network structure of a scientific research team.

**FIGURE 3:**
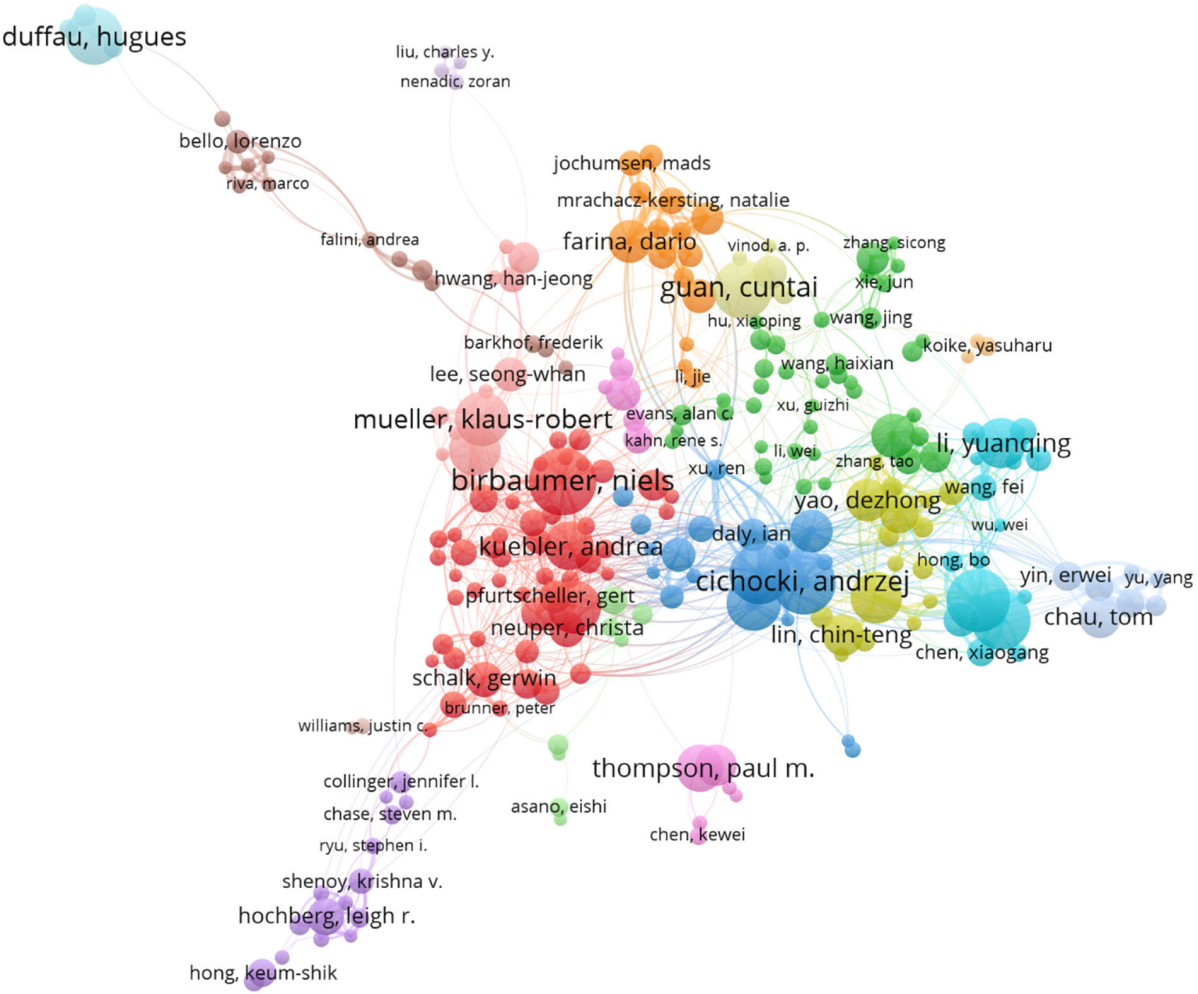
Author's cooperation map. According to programmed calculations and a two‐dimensional diagram. There are 40,897 authors in total, of whom 2094 have collaborative relationships, with a minimum value set at 14.

According to programmed calculations and a two‐dimensional diagram, there are 40,897 authors in total, of which 2094 have collaborative relationships, with the minimum value set at 14. Among these, 296 authors formed a collaborative network (Figure 3). Niels Birbaumer, Andrzej Cichocki, Yijun Wang, Jing Jin, and Gao Xiaorong stand out among many researchers, and the overall research team shows a high degree of separation and a low degree of cohesion. Collaboration among researchers is mainly concentrated within their own institutions, and cross‐national group cooperation has not yet been formed. There is little exchange and interaction among different groups, and the overall situation is characterized by “separation.” According to the model data, in recent years, there has been a trend in team‐oriented construction in brain science research, with Niels Birbaumer, Andrea Kuebler, Gernot R. Mueller‐Putz, and Gerwin Schalk as the core team, Andrzej Cichocki and Yu Zhang as the central team, and Yijun Wang, Gao Xiaorong, and Yuanqing Li as the central team. There is indirect cooperation between these teams, whereas the team of Leigh R. Hochberg, Hugues Duffau, and Arthur W. Toga demonstrates only close internal cooperation.

Through statistical analysis of the authors of published articles, we can identify the core authors in the field of brain science research. Based on the number of published articles, the top five authors in the past 20 years are Niels Birbaumer from Eberhard Karls University of Tubingen (with 93 articles), Andrzej Cichocki from Skolkovo Institute of Science and Technology (Skoltech) (with 86 articles), Yijun Wang from the Institute of Semiconductors (with 86 articles), and Jing Jin from Tsinghua University (with 81 articles) (Table 2). Among the highly prolific authors, the number of published papers is generally concentrated between 70 and 90, while the top author has published 93 articles.

**TABLE 2:** Top 10 active authors with the most documents.

Ranking	Authors	Documents	Citations	Organizations	Country	First year
1	Niels Birbaumer	93	8594	Eberhard Karls University of Tubingen	Germany	2006
2	Andrzej Cichocki	86	5006	Skolkovo Institute of Science and Technology (Skoltech)	USA	2006
2	Yijun Wang	86	5209	Institute of Semiconductors	China	2006
4	Jing Jin	81	4317	Tsinghua University	China	2006
5	Gao Xiaorong	79	5601	Tsinghua University	China	2006
6	Cuntai Guan	79	6068	Nanyang Technological University	China	2006
7	Hugues Duffau	76	5523	Universite de Montpellier	France	2006
8	Klaus‐Robert Müller	72	9732	Technische Universitaet Berlin	Germany	2006
9	Xingyu Wang	71	4056	University of Science and Technology of China	China	2010
10	Gernot Müller‐Putz	70	3494	Graz University of Technology	Austria	2005

### 3.2 Gradual Centralization of Brain Science Research Source Institutions

The network of national and institutional collaborations in brain science research is calculated by associating the Cite Space operational with the network model “PF‐NET,” setting Nodes (TopN, e) = {n(i), TopN = 50, TopN% = 10%, and the literature monolith with “g2 ≤ kΣi ≤ GCI, k ∈ z+, k = 25” as the interval value.

#### 3.2.1 High Prominence Country Cooperation

**FIGURE 4:**
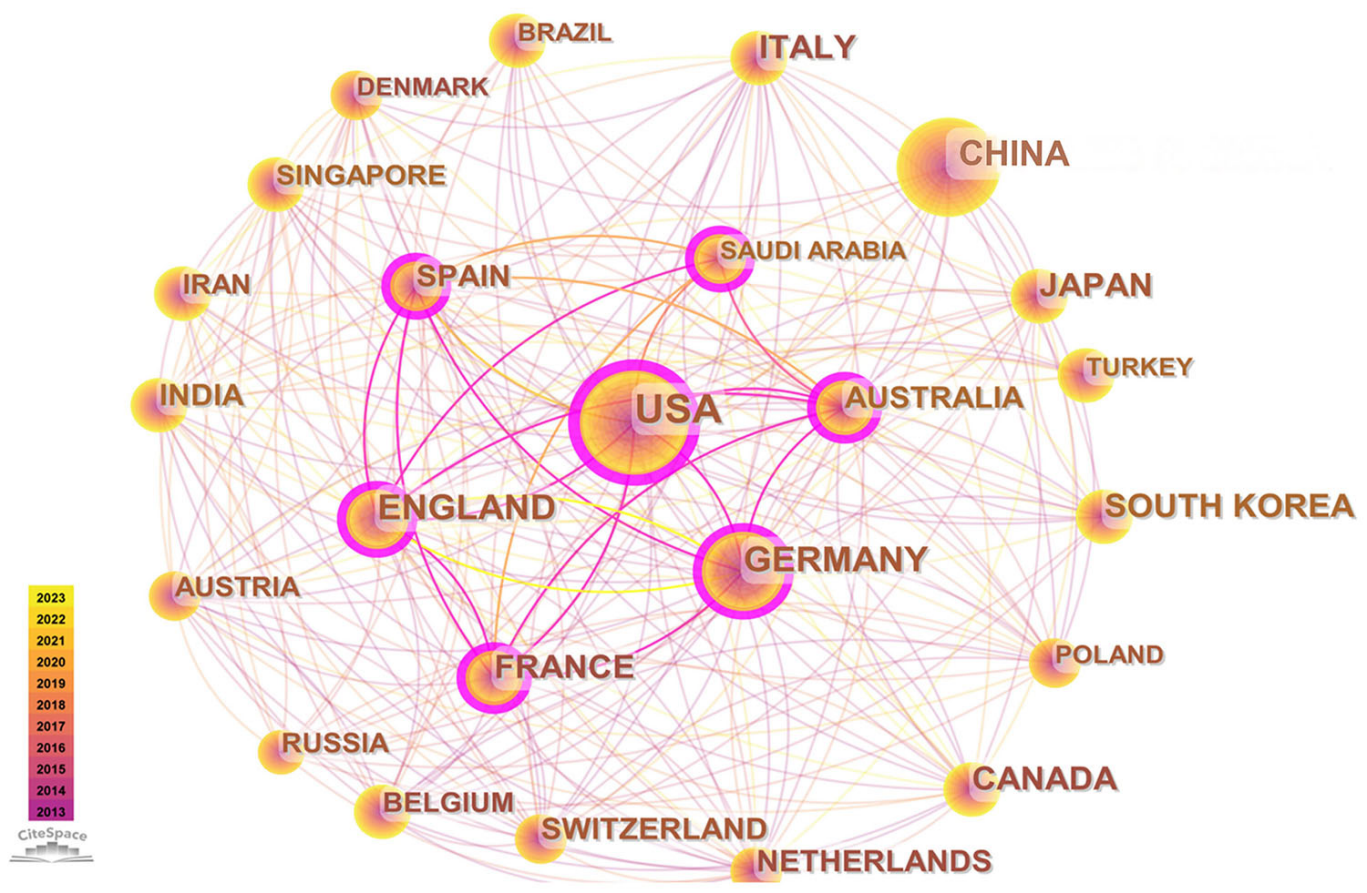
Cross‐country collaborations visualization map. The network of cross‐country collaborations of brain science research is calculated by associating the Cite Space operational with the network model “PF‐NET,” setting Nodes (TopN, e) = {n(i), TopN = 50, TopN% = 10%, and the literature monolith with “g2 ≤ kΣi ≤ GCI, k ∈ z+, k = 25” as the interval value.

The program data show that the network: *N* = 105 and *E* = 881, a total of 105 countries and regions have carried out brain science research over the past decade, with the United States, United Kingdom, France, and Austria forming the primary cooperative group. Cooperation is active, and China has the highest number of publications. However, relatively little international collaboration was observed (Figure 4).

#### 3.2.2 More Centralized Connected Network Clusters

**FIGURE 5:**
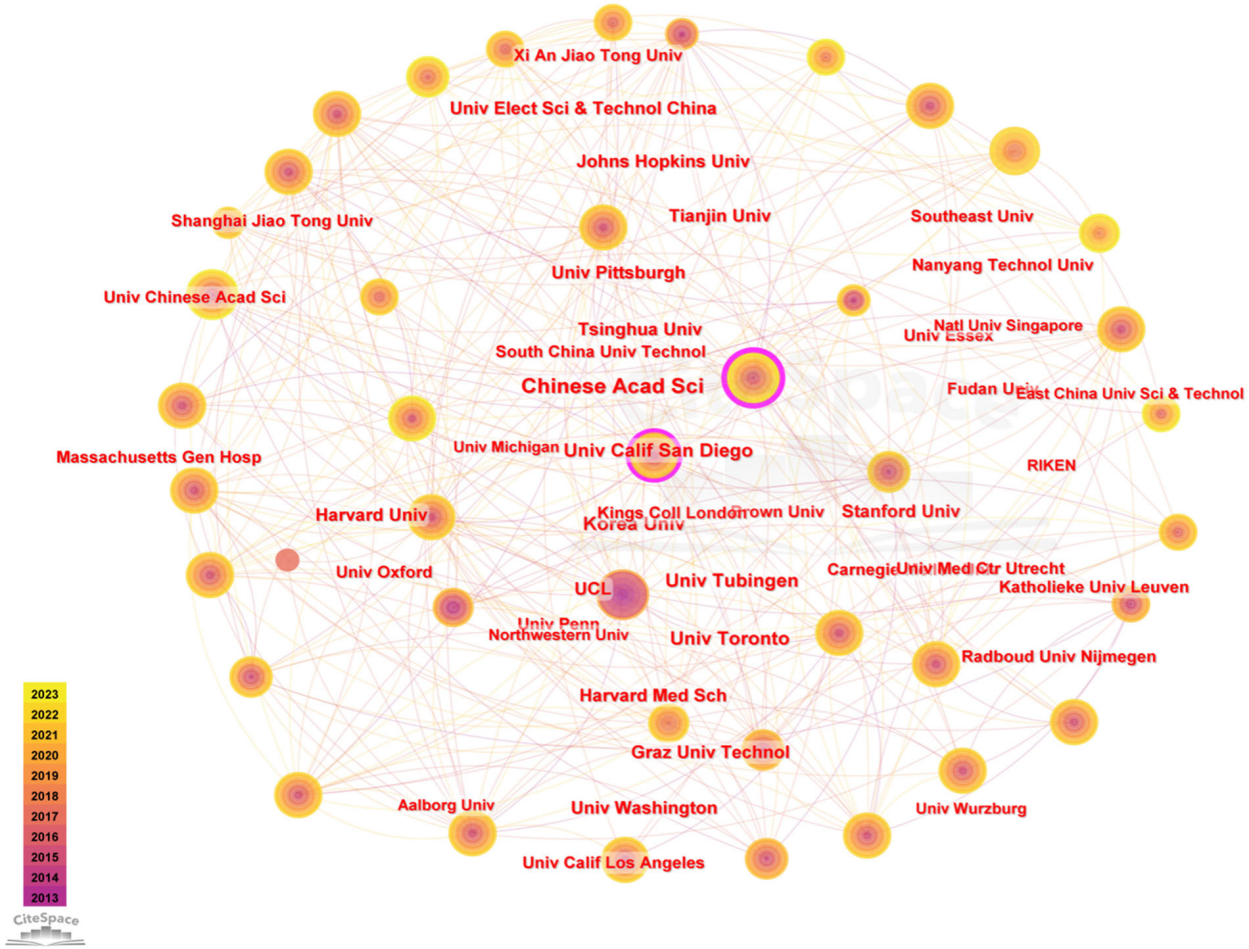
Institutional cooperation map. The network of institutional collaborations of brain science research is calculated by associating the Cite Space operational with the network model “PF‐NET,” setting Nodes (TopN, e) = {n(i), TopN = 50, TopN% = 10%, and the literature monolith with “g2 ≤ kΣi ≤ GCI, k ∈ z+, k = 25” as the interval value.

The program data showed that the network had *N* = 560 and *E* = 3703. In the past decade, 560 research institutions have conducted brain science research, mainly led by renowned universities. Universities are the main force of global brain science research. From the most critical institutional cooperation map (Figure 5), the Chinese Academy of Sciences in China, the University of California, San Diego in the United States, and University College London in England have the highest prominence in brain science research. The Chinese Academy of Sciences, ranked first, has a centrality of 0.2, followed by the University of California San Diego with a centrality of 0.11 and UCL in England with a centrality of 0.1. However, the four research institutions, Korea University in Korea, the University of Pittsburgh in the United States, Tsinghua University, and Tianjin University in China, have a high publication output and low centrality. These data indicate that their research collaboration with the outside world may still require further strengthening to better integrate into the global brain science research network (Table 3).

**TABLE 3:** The distribution of the top 10 countries/regions and institutions by the number of brain science publications.

Ranking	Country/territory	Frequency	Centrality	Ranking	Institution	Frequency	Centrality	Country
1	USA	2516	0.27	1	Chinese Academy of Sciences	253	0.2	China
2	China	2080	0.06	2	University of California San Diego	160	0.11	USA
3	Germany	1069	0.12	3	University of Tubingen	156	0.07	Germany
4	England	710	0.13	4	Korea University	133	0.03	Korea
5	Canada	521	0.08	5	University of Toronto	128	0.07	Canada
6	Italy	510	0.03	6	Tsinghua University	125	0.04	China
7	France	483	0.18	7	University of Pittsburgh	120	0.02	USA
8	South Korea	476	0.02	8	Stanford University	117	0.08	USA
9	Japan	467	0.05	9	Tianjin University	112	0.02	China
10	Spain	363	0.12	10	UCL	109	0.1	England

### 3.3 Journal and Literature Citation Analysis

#### 3.3.1 Journal Citations Analysis

A total of 192 journals contributed to the publication of brain science research. Over the past decade, the top five journals are “Human Brain Mapping” (1364 papers), “Journal of Neural Engineering” (590 papers), “Frontiers in Brain Science Research” (341 papers), “Ieee Transactions on Neural Systems and Rehabilitation Engineering” (328 papers), and “Frontiers in Human Brain Science Research” (312 papers). These journals have published important articles on brain science research, particularly “Human Brain Mapping,” which not only has the highest publication volume but is also ranked in the JCR subject category Q1 zone and has a higher impact factor, making it the most important journal in the field of brain science research (Table 4). In terms of the number, frequency, and centrality of citations, high‐impact factor journals such as “PNAS,” “NeuroImage,” and “Journal of Neural Engineering” are quite active in this field. Although they do not have many publications, they do have many collaborative citations. This means that the quality of their publications was far above the average (Table 5).

**TABLE 4:** Top 10 journals with the largest number of publications.

Ranking	Journals	Publications	2022 impact factor	2022 JCR partition
1	Human Brain Mapping	1364	5.399	Q1
2	Journal of Neural Engineering	590	5.043	Q2
3	Frontiers in Neuroscience	341	5.152	Q2
4	IEEE Transactions on Neural Systems and Rehabilitation Engineering	328	4.528	Q2
5	Frontiers in Human Neuroscience	312	3.473	Q3
6	Sensors	233	3.847	Q2
7	Biomedical Signal Processing and Control	223	5.076	Q2
8	IEEE Access	215	3.476	Q2
9	Plos One	175	3.752	Q2
10	NeuroImage	166	7.400	Q1

**TABLE 5:** Top 10 Cited‐journals with the largest number of frequency.

Ranking	Cited‐journals	Frequency	Centrality	2022 impact factor	2022 JCR partition
1	NeuroImage	5415	0.11	7.400	Q1
2	Journal of Neural Engineering	4643	0.07	5.043	Q2
3	Plos One	4306	0.05	3.752	Q2
4	Clinical Neurophysiology	4206	0.02	4.861	Q2
5	IEEE Transactions on Biomedical Engineering	4169	0.03	4.756	Q2
6	IEEE Transactions on Neural Systems and Rehabilitation Engineering	3964	0.06	4.528	Q2
7	Journal of Neuroscience And Methods	3353	0.01	2.987	Q3
8	Proceedings of the National Academy of Sciences of The United States of America (PNAS)	3295	0.02	9.412	Q1
9	Journal of Neuroscience	3156	0.04	6.709	Q1
10	Frontiers in Neuroscience	3009	0.05	5.152	Q2

#### 3.3.2 Literature Citations and Highly Cited Literature Analysis

References in an article demonstrate the continuity of scientific research and provide a scientific basis for the author's argument. We have summarized the references cited in articles published over the past decade. Table 6 shows the top 10 cited articles, of which nine are research papers, and one is a review article. The citation frequency of literature reflects the value of literature in the field of brain science research over the past decade, and highly cited papers constitute the knowledge foundation of brain science research. They not only provide a theoretical basis for further research at the forefront of brain science research technology but also lay the foundation for the application of brain science research scenarios. It is found that the paper “Deep Learning With Convolutional Neural Networks for EEG Decoding and Visualization” by Schirrmeister Robin Tibor has an important role in the field both as an article with high citations and as a highly cited article in the field (Tables 6 and 7). This indicates that Schirrmeister Robin Tibor and their Translate Neurotechnol Lab at the University of Freiburg may be the most promising and powerful innovation sources in the field of brain science research in the future.

**TABLE 6:** The top 10 cited literature items on “brain science.”

Ranking	Title	Type	Total citations	Centrality	Publication year	Citation	Author
1	Deep Learning With Convolutional Neural Networks for EEG Decoding and Visualization	Article	301	0.05	2017	Hum Brain Mapp, V38, P5391, DOI 10.1002/hbm.23730	Schirrmeister RT
2	EEGNet: A Compact Convolutional Network for EEG‐based Brain–computer Interfaces	Article	291	0.02	2018	J Neural Eng, V15, P0, DOI 10.1088/1741‐2552/aace8c	Lawhern VJ
3	A Review of Classification Algorithms for EEG‐based Brain–computer Interfaces: A 10‐year Update	Review	283	0.05	2018	J Neural Eng, V15, P0, DOI 10.1088/1741‐2552/aab2f2	Lotte F
4	Enhancing Detection of SSVEPs for a High‐Speed Brain Speller Using Task‐Related Component Analysis	Article	207	0.06	2018	IEEE T Bio‐Med Eng, V65, P104, DOI 10.1109/TBME.2017.2694818	Nakanishi M
5	A novel deep learning approach for classification of EEG motor imagery signals	Article	188	0.05	2017	J Neural Eng, V14, P0, DOI 10.1088/1741‐2560/14/1/016003	Tabar YR
6	inverse reinforcement learning to control a robotic arm using a brain–computer interface inverse reinforcement learning to control a robotic arm using a brain–computer interface	Article	176	0.02	2012	Nature, V485, P372, DOI 10.1038/nature11076	Hochberg LR
7	High‐speed spelling with a noninvasive brain–computer interface.	Article	169	0.03	2015	P Natl Acad Sci USA, V112, PE6058, DOI 10.1073/pnas.1508080112	Chen XG
8	High‐performance neuroprosthetic control by an individual with tetraplegia	Article	168	0.09	2013	Lancet, V381, P557, DOI 10.1016/S0140‐6736(12)61816‐9	Collinger JL
9	Brain–computer interfaces for communication and rehabilitation	Article	164	0.05	2016	Nat Rev Neurol, V12, P513, DOI 10.1038/nrneurol.2016.113	Chaudhary U
10	Brain–machine interface in chronic stroke rehabilitation: a controlled study.	Article	145	0.05	2013	Ann Neurol, V74, P100, DOI 10.1002/ana.23879	Ramos‐Murguialday A

**TABLE 7:** Top 10 citation analysis of documents on “brain science.”

Ranking	Title	Type	First author	Source	Publication year	Total citations	Main content
1	Deep Learning With Convolutional Neural Networks for EEG Decoding and Visualization	Article	Schirrmeister, Robin Tibor	Human Brain Mapping	2017	941	Our study thus shows how to design and train ConvNets to decode task‐related information from the raw EEG without handcrafted features and highlights the potential of deep ConvNets combined with advanced visualization techniques for EEG‐based brain mapping.
2	EEGNet: A Compact Convolutional Neural Network for Eeg‐Based Brain–Computer Interfaces	Article	Lawhern, Vernon J.	Journal of Neural Engineering	2018	685	In this work, we introduce EEGNet, a compact convolutional neural network for EEG‐based BCIs. We introduce the use of depthwise and separable convolutions to construct an EEG‐specific model that encapsulates well‐known EEG feature extraction concepts for BCI.
3	On the Interpretation of Weight Vectors of Linear Models in Multivariate Neuroimaging	Article	Haufe, Stefan	NeuroImage	2014	634	On the interpretation of weight vectors of linear models in multivariate neuroimaging. We hope that this work raises awareness for an often encountered problem and provides a theoretical basis for conducting better interpretable multivariate neuroimaging analyses.
4	A Review of Classification Algorithms for EEG‐Based Brain–Computer Interfaces: A 10‐year Update	Review	Lotte, F.	Journal of Neural Engineering	2018	626	This paper provides a comprehensive overview of the modern classification algorithms used in EEG‐based BCIs, presents the principles of these methods and provides guidelines on when and how to use them. It also identifies a number of challenges to further advance EEG classification in BCI.
5	Investigating the Prevalence of Complex Fiber Configurations in White Matter Tissue With Diffusion Magnetic Resonance Imaging	Article	Jeurissen, Ben	Human Brain Mapping	2013	604	Investigating the prevalence of complex fiber configurations in white matter tissue with diffusion magnetic resonance imaging.
6	The extraction of Neural Information From the Surface EMG for the Control of Upper‐Limb Prostheses: Emerging Avenues and Challenges	Article	Farina, Dario	IEEE Transactions on Neural Systems and Rehabilitation Engineering	2014	534	Motor unit myoelectric control neural drive to muscle pattern recognition regression
7	Resting‐State Networks Show Dynamic Functional Connectivity in Awake Humans and Anesthetized Macaques	Article	Hutchison, R. Matthew	Human Brain Mapping	2013	502	We characterize RSN dynamics of anesthetized macaques that control for these accounts and compare them to awake human subjects.
8	Clarity for Mapping the Nervous System	Article	Chung, Kwanghun	Nature Methods	2013	479	Clarity for mapping the nervous system
9	Deep Learning for Healthcare Applications Based on Physiological Signals: A Review	Review	Faust, Oliver	Computer Methods and Programs in Biomedicine	2018	471	This review paper depicts the application of various deep learning algorithms used till recently, but in the future it will be used for more healthcare areas to improve the quality of diagnosis.
10	The Grand Challenges of Science Robotics	Article	Yang, Guang‐Zhong	Science Robotics	2018	467	Brain–computer interfaces legged locomotion design, soft cognition, fabrication machine, trends, future

### 3.4 The Evolution of Topics in Brain Science Research

**FIGURE 6:**
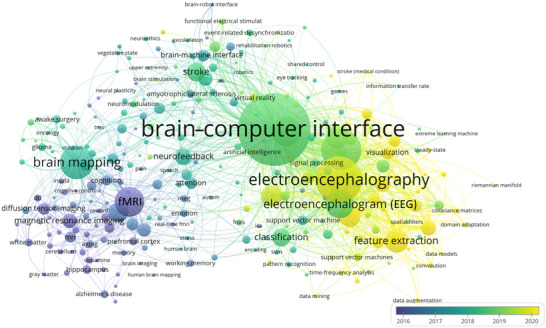
Co‐occurrence map of keywords. In keyword co‐occurrence analysis, there are a total of 300 keywords that are limited to appearing no less than 14 times. The larger the keyword size of the same type, the higher the frequency of occurrence, and the color represents the time period of keyword occurrence.

Keywords play a crucial role in highlighting the core of research topics. Unitizing the collective data of brain science research helps clarify the research theme, grasp research hotspots, and understand the progress in brain science research over the past decade. By presenting a co‐occurrence heat map sorted by the level of heat, we can identify the top keywords that represent different focal points of brain science research, including “functional magnetic resonance imaging (fMRI),” “brain mapping,” “brain–computer interface (BCI),” “electroencephalography,” and “feature extraction.” To better understand the research level and historical development dynamics of the field of brain science research and to clarify its static structural system, the obtained keywords were presented in the time dimension using a progressive graph. From the analysis of high‐frequency keywords (Table 8) and the keyword co‐occurrence network (Figure 6), it is found that in 2016, the hotspots were concentrated in the field of fMRI. Many neuroscientists have begun to engage in fMRI research and apply it to cognitive brain science research. The urgent need in the medical field has further promoted the development of fMRI technology, and some applications in pathology have begun to emerge, such as the use of diffusion imaging and perfusion imaging techniques to diagnose local cerebral ischemia. In 2017, “brain mapping” became a research hot topic, and with the continuous maturity and iteration of fMRI technology, “brain mapping” technology has been applied to clinical practice. In 2018, BCIs became a hot research topic. The hot research topics in the past 2 years have been “electroencephalography” and “feature extraction.” The following are the most popular research in those years: Gupta et al. (2022) identified multiple cognitive tasks from EEG signals using the EMD method. This paper presents a novel feature representation (formation of the most informative features) of the EEG signal for both binary and multi‐MTC using a combination of statistical, uncertainty, and memory‐based coefficients. Cao et al. (2022) proposed a new EEG‐based connectivity network analysis feature selection based on the BCI for motor modalities that can facilitate neurorehabilitation in stroke patients, which is important for developing an effective BCI training program for stroke rehabilitation. Wu et al. (2022) proposed a four‐dimensional brain mapping method that can represent the continuous process of the human fatigue state in the spatiotemporal domain as image frames and can be applied to any type of brain fatigue detection. Algarni et al. (2022) presented a method based on deep learning for the emotion recognition of EEG signals, which includes four stages: data selection, feature extraction, feature selection, and classification. This research contributes to improving the performance of emotion recognition models to obtain more accurate results, thus helping in making correct medical decisions. Singanamalla and Lin (2022) designed a method that uses a spiking neural network (SNN), which is trained using agent gradient descent to generate task‐related and multichannel EEG template signals for all categories. The proposed method compares the classification performance of the EEG signals and their spike representations. Naive Bayes was used to improve the performance of the ERN dataset from 79.22% to 82.27%, whereas xGBoost was used to increase the accuracy of the P300 dataset from 67.73% to 69.87%. Rungsirisilp and Wongsawat (2022) applied joint action observation (AO) and motion imagery (MI) to improve the classification performance of a brain–machine interface system for stroke patients. Under conditions of MI combined with AO, it is more effective in enhancing event‐related desynchronization (ERD) than MI or AO alone. Khoshnevis and Sankar (2022) used new higher‐order statistical (HOS) features of EEG signals in alpha and beta rhythms to diagnose Parkinson's disease. This machine learning method improves the classification results by combining multiple models and produces better prediction performance.

**TABLE 8:** High‐frequency keywords.

Rank	Keywords	Frequency	Rank	Keywords	Frequency
1	brain–computer interface	1508	11	p300	218
2	electroencephalogram (EEG)	595	12	stroke	207
3	electroencephalography	786	13	classification	194
4	motor imagery	637	14	neurofeedback	169
5	brain mapping	488	15	task analysis	160
6	fMRI	406	16	brain modeling	150
7	feature extraction	289	17	Magnetic resonance imaging	140
8	deep learning	260	18	rehabilitation	136
9	machine learning	245	19	functional magnetic resonance imaging	132
10	functional connectivity	218	20	transfer learning	121

### 3.5 Hotspot Prospects of Topic Clustering and Temporal Evolution Based on Data Models

**FIGURE 7:**
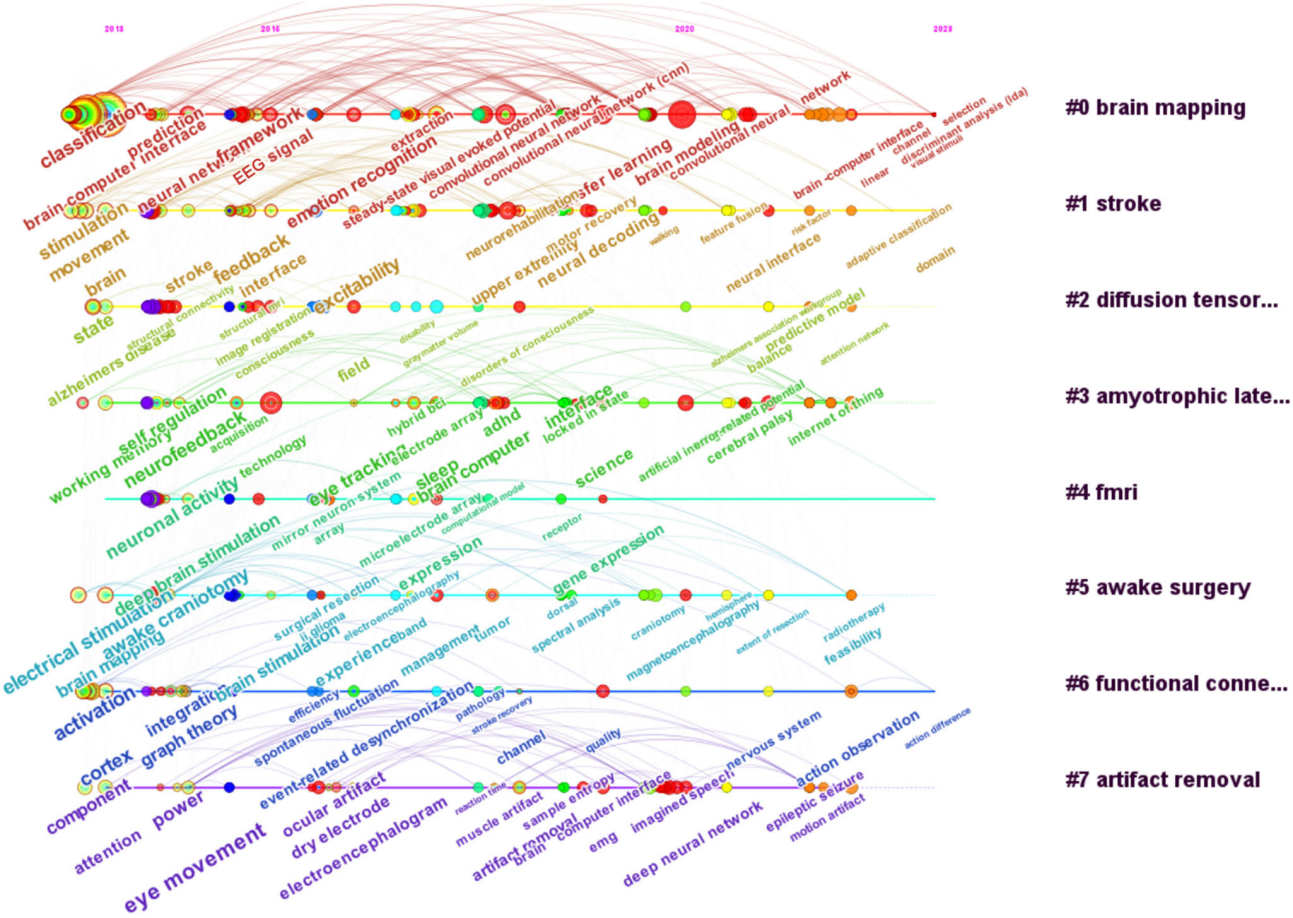
Timeline view of reference clustering. Nodes represent keywords (the bigger the circle, the more frequently it appears), and the labels of the clusters are listed on the right, where #2 is diffusion tensor imaging, #3 is amyotrophic lateral sclerosis, and #6 is functional connectivity.

Based on the evolution of keywords in brain science research, clustering was performed using the similarity measurement of LLR in the association operation rules. Topic clustering and temporal evolution are presented using the timeline model under clustering, forming a cluster group to capture the dynamic changes in brain science research and explore its trends and hotspots. By analyzing the hot topics, application areas, and evolutionary trends in the field of brain science research, we identified the top eight research clusters with the highest attention: #0 brain mapping, #1 stroke, #2 diffusion tensor imaging, #3 amyotrophic lateral sclerosis, #4 fMRI, #5 awake surgery, #6 functional connectivity, and #7 artifact removal (Figure 7 and Table 9). The degree of connection between research clusters in the field of brain science research varies and involves a relatively wide range of areas. Amyotrophic lateral sclerosis (#3), fMRI (#4), and awake surgery (#5) studies, as well as functional connectivity (#6) and artifact removal (#7) studies, have some overlap, indicating a common phenomenon of co‐citation between research clusters. Based on the research directions obtained from the co‐occurrence of keywords, we conducted a secondary reading of the literature included in each research cluster and merged similar directions to identify three hot research areas in brain science research: brain exploration, brain protection, and brain creation.

**TABLE 9:** Keyword cluster analysis.

Cluster ID	Clustering theme	Top terms
#0	Brain mapping	Classification; prediction; brain–computer interface; framework; EEG signal; extraction; emotion recognition; convolutional neural network; transfer learning; brain modeling; channel selection
#1	Stroke	Stimulation; movement; excitability; neural decoding; neural interface; adaptation classification
#2	diffusion tensor imaging	State; image registration; disability; field; disorders of consciousness; predictive model; balance; attention network
#3	Amyotrophic lateral sclerosis	Self‐regulation; working memory; neurofeedback; acquisition; hybrid BCI; eye tracking; electrode array; sleep; brain–computer interface; cerebral palsy; Internet of Thing
#4	fMRI	neuronal activity; deep brain stimulation; microelectrode array; receptor; gene expression
#5	Awake surgery	electrical stimulation; awake craniotomy; brain stimulation; experience; management; spectral analysis; magnetoencephalography; radiotherapy; feasibility
#6	Functional connectivity	Activation; cortex; integration; graph theory; spontaneous fluctuation; event‐related desynchronization; channel; quality; nervous system; action observation
#7	Artifact removal	Component; attention; power; eye movement; ocular artifact; dry electrode; electroencephalogram; muscle artifact; artifact removal; EMG; imagined speech; deep neural network

#### 3.5.1 “Brain Exploration,” the Exploration of the Function, Structure, and Principles of the Brain, Including the Physical Composition, Biological Mechanisms, and Working Functions of the Brain

Cluster #2 (diffusion tensor imaging), Cluster #4 (fMRI), and Cluster #7 (artifact removal) were applied to brain exploration and cognitive research (Figure 7). In terms of brain development, Cortina et al. (2021) used magnetic resonance relaxation and diffusion tensor imaging to explore gender and age‐related microstructural differences in brain white matter. Bernard et al. (2022) discovered a synaptic signaling pathway for controlling the excitability of pyramidal cells and inhibitory interneurons expressing parvalbumin, providing the first evidence for protein synthesis regulation specificity in brain development. In terms of perception, researchers used optogenetic techniques to manipulate the ventral–posterior–medial thalamus of the tactile pathway in awake mice and measured the discharge activity of the thalamus and primary somatosensory cortex using extracellular electrophysiology and genetically encoded voltage imaging techniques (Borden et al. 2022).

In terms of learning and memory, researchers have inferred the possible existence of a retrograde mechanism that coordinates the aggregation of memory neurons in different brain regions (Lavi et al. 2023). Cai et al. (2021) confirmed that the difference in cortical thickness between mild cognitive impairment and degeneration is related to the upregulation of genes involved in chemical synaptic transmission. Tsai et al. (2022) proposed an adaptive online automatic speech recognition (ASR) technique that integrates Hebbian/anti‐Hebbian neural networks into the ASR algorithm based on the adaptive pseudo shadow subspace reconstruction technique of Hebbian/anti‐Hebbian learning networks to enhance the performance of brain–machine interfaces.

#### 3.5.2 “Brain Protection,” Treatment of Brain‐Related Diseases

Scientists are attempting to translate these discoveries into breakthroughs in treating human physiological and psychological ailments by gaining a deeper understanding of brain mechanisms and processes. Clusters #1 (stroke), #3 (amyotrophic lateral sclerosis), and #5 (awake surgery) (Figure 7) represent applications of brain science research in the field of disease treatment. Stroke and amyotrophic lateral sclerosis remain challenges in the urgent need for breakthroughs in brain science research. Bermudez I Badia et al. (2013) proposed in 2013 a hybrid BCI‐virtual reality (VR) system that combines personalized motor training in a VR environment, exploiting brain mechanisms for action execution and observation, and a neurofeedback paradigm using mental imagery as a way to engage secondary or indirect pathways to access undamaged corticospinal tracts. Subsequently, BCI, MI‐based BCI training, and MI and VR augmentation‐based BCI training have been adopted to promote the recovery of upper limb function, cortical excitability, and cognitive task performance in stroke patients (Tao et al. 2023, Pichiorri et al. 2023, Kern et al. 2022, Aristela de Freitas et al. 2022). In 2014, Goljahani et al. (2014) proposed that preprocessing based on Bayesian single‐trial event‐related potential estimation technology provided feasibility for P300‐based single‐channel BCIs to improve operability and acceptability for patients with amyotrophic lateral sclerosis. In recent years, artificial intelligence technologies, such as interpretable convolutional neural networks and deep learning, have been used in BCIs to aid the movement of patients with amyotrophic lateral sclerosis. The brain–machine interface speller that encodes code‐modulated visual evoked potentials (cVEPs) can enhance communication with patients with amyotrophic lateral sclerosis (Riccio et al. 2022, Aliakbaryhosseinabadi et al. 2021, Verbaarschot et al. 2021, Tajmirriahi et al. 2022). In 2013, Silva Paiva et al. (2013) described an approach guided by frameless neuronal activation and preoperative functional mapping with transcranial magnetic stimulation (TMS) for surgical planning. They verified complete control of seizures (Engel class 1A) in patients with refractory epilepsy. T. Li et al. (2015) used direct electrical stimulation for awake surgery for the localization and removal of gliomas responsible for language mapping. Direct electrical stimulation has been proven to be a reliable and noninvasive method for intraoperative language mapping, allowing for the safe removal of language areas in gliomas. Motomura et al. (2015) evaluated research showing that awake mapping can protect higher cognitive functions, including working memory and spatial cognition, in patients with nondominant right frontal lobe tumors. However, due to insufficient knowledge of the etiology and development of many brain diseases, many of them remain incurable.

#### 3.5.3 “Brain Creation,” the Development of Brain Functions or the Borrowing of Brain Function Development Techniques

With a precise understanding of the brain's function and operational mechanisms, humans have begun to utilize and enhance the brain's capabilities in multiple ways, even by creating artificial brains. Clusters #0 (brain mapping) and #6 (functional connectivity) (Figure 7) were aimed at developing and applying brain functions. There are three main directions. First, in‐depth research on brain–machine interface technology is conducted to build seamless, real‐time, and side‐effect‐free brain–machine interfaces and enable perfect bidirectional communication between the brain and machines by timely recording and interpreting neural interaction patterns. In 2013, Rezakova et al. (2013) conducted research on dynamic mapping of the brain and cognitive control of virtual games using fMRI technology. Subsequently, new technologies such as extraction, emotion recognition, convolutional neural networks, transfer learning, brain modeling, and channel selection have been applied to the development of brain–machine interfaces (Luckett et al. 2023, Jami et al. 2022, Blood 2022, J. Zhang, Liu, et al. 2022).

In addition, achieving direct communication between the brain and machines has become a new path for enhancing human capabilities. Through technologies such as AR and VR, BCIs can assist in neural rehabilitation, create virtual sensations, and improve brain performance in learning, memory, attention, and other aspects. For example, Xu et al. (2023) proposed a framework for dividing sleep stages using multi‐subsegment functional brain connections and utilized phase‐locked values to construct brain networks to explore the mechanisms of brain functional connections. They will use graph convolutional networks for automatic sleep stage monitoring and develop an online BCI system, which has important potential for improving the application of sleep staging.

Third, research on neural mechanisms, cognitive structures, and brain–machine interfaces can contribute to breakthroughs in the field of artificial intelligence, leading to the design of new learning models that can mimic human brain neuron activity and ultimately lead to the development of more intelligent machines and robots. Huang et al. (2022) proposed an active learning framework called variational deep embedding‐based active learning (VaDEAL) as a human‐centric computing method to improve the accuracy of diagnosing pneumonia. S. G. Zhang, Chen, Zhang, et al. (2022) introduced augmented reality (AR) technology to present visual stimuli for SSVEP‐BCI (BCI based on steady‐state visual evoked potentials) and designed a robot grasping experiment to validate the applicability of the AR‐BCI system. The results showed that the AR‐SSVEP‐NAO system was applicable to robot‐grasping tasks. Technologies such as AR, novel imaging, neural monitoring, and regulation will promote rapid development in various subfields of brain exploration, protection, and creation. Neuromorphic computing and brain–machine intelligence will transition from “inspired by brain structure” to “balancing inspiration from brain structure and function,” develop “perception intelligence” and “cognitive intelligence” synergistically, and shift from “specialized intelligence” to “general intelligence.”

**FIGURE 8:**
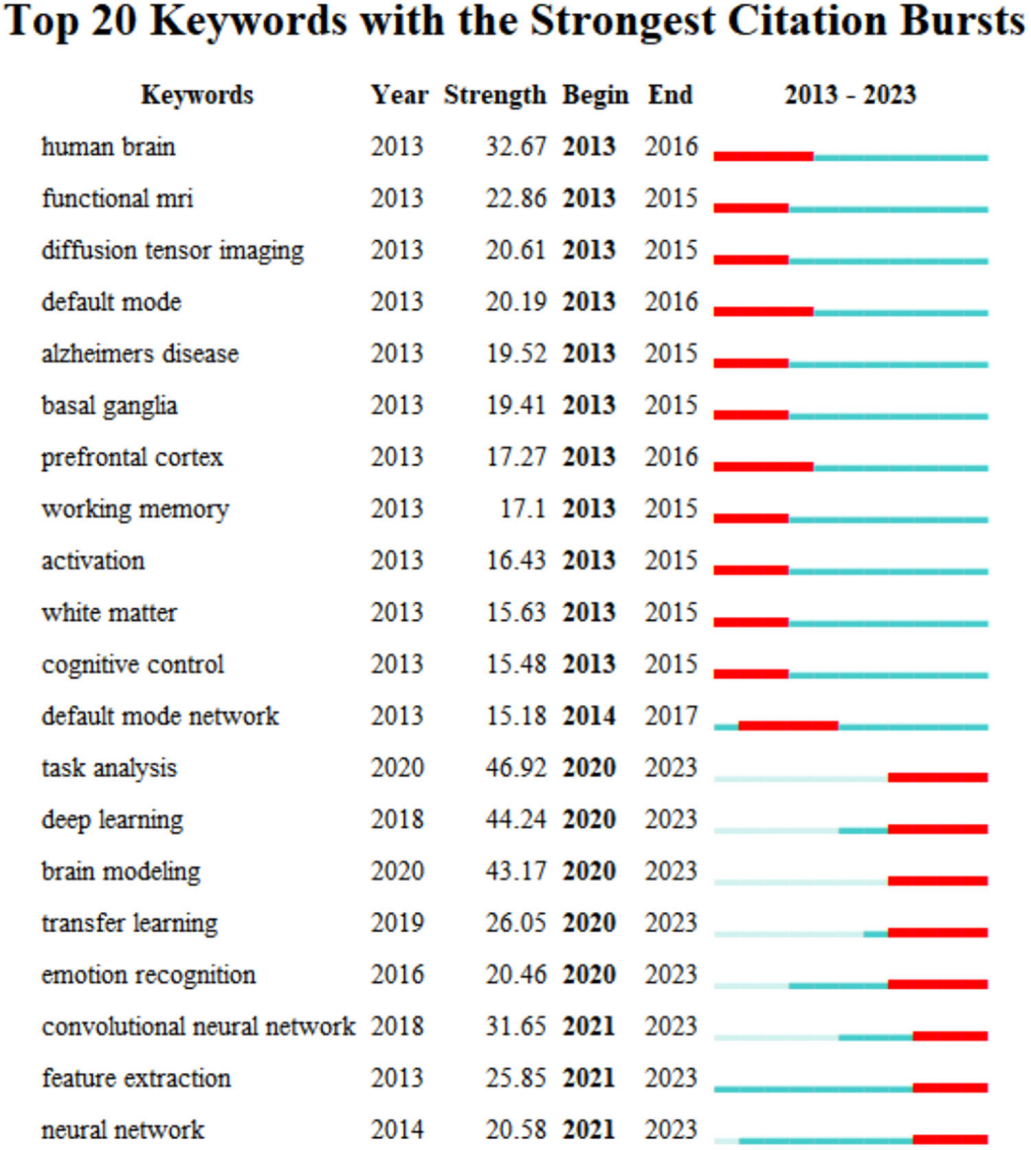
Keyword emergence analysis in the literature. A total of 20 emergent words are detected in the literature analysis of brain science.

Keyword co‐occurrence analysis is based on the detection of keywords with high frequency and rapid growth within a certain period of time. Keywords with high burst intensity are important indicators that reflect research hotspots, frontiers, and the latest trends. Twenty emergent words were identified (Figure 8). “Task analysis” had the highest burst intensity, reaching 46.92, and occurred between 2020 and 2023. “Deep learning” was the second highest, occurring between 2020 and 2023. Both are important research topics and applications in the field of machine learning. The early explosive growth of keyword citations mainly focused on brain disorders and cognitive control. With further research, research hotspots have become more diverse. Since 2018, brain‐like artificial intelligence has emerged in the field of brain science research. Underlying theories and algorithm models in the field of artificial intelligence, such as “task analysis,” “deep learning,” “transfer learning,” “feature extraction,” and “neural network,” are gradually increasing, becoming the frontier and hot topic of current research in the field of brain science research. This explains the main source of China's recent progress in the field of brain science research: the rapid breakthrough in the field of scientific research driven by the extensive application of artificial intelligence and the use of advanced technologies such as artificial intelligence and big data algorithms to accelerate the filling of gaps in traditional brain science fields such as neural science, thus achieving an important achievement in the number of papers published, rising from sixth to second place globally in the past decade. The noteworthy points are the explosive references of keywords such as “task analysis” (2020–2023, 49.92), “deep learning” (2020–2023, 44.24), “brain modeling” (2020–2023, 43.17), “transfer learning” (2020–2023, 26.05), “emotion recognition” (2020–2023, 20.46), “convolutional neural network” (2021–2023, 31.65), “feature extraction” (2021–2023, 25.85), “neural network” (2021–2023, 20.58), and so on, which will continue to be the future research hotspots, as indicated by their sustained prevalence until 2023.

### 3.6 Overview of Global Competitiveness in Brain Science Research

**FIGURE 9:**
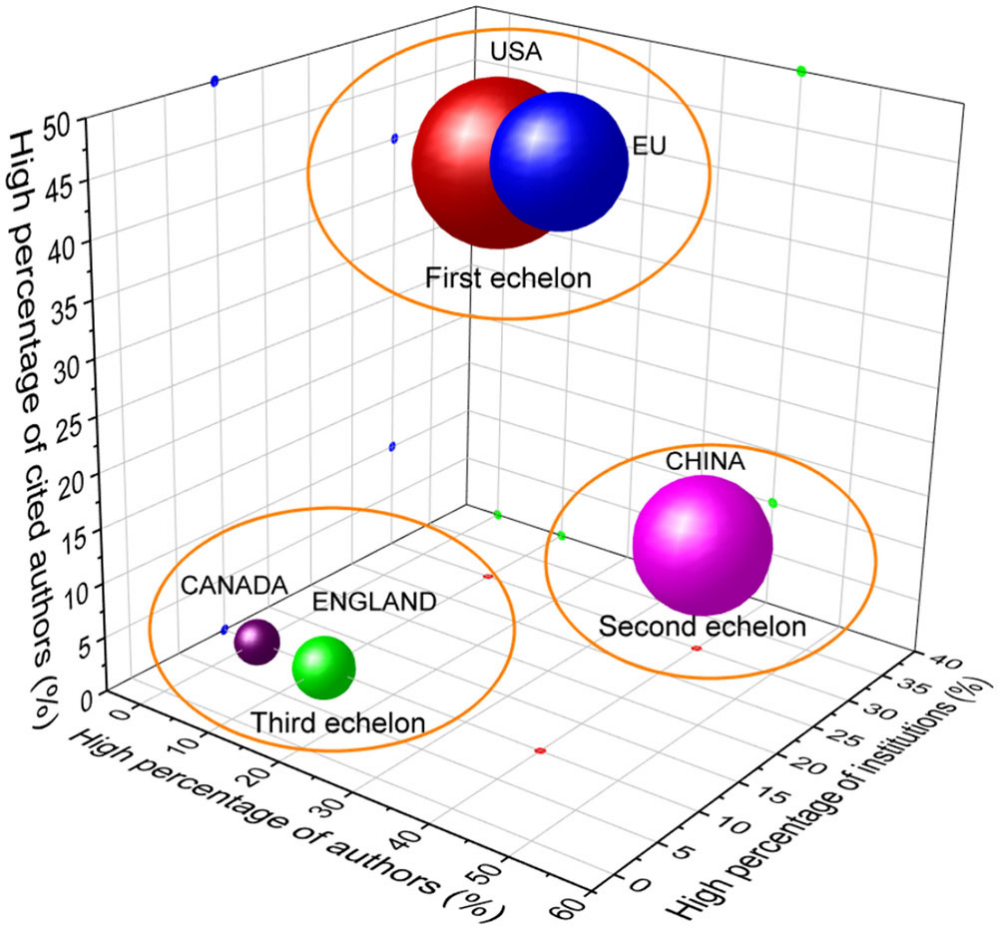
Competitiveness of brain science research in major countries/organizations. The bubble volume is determined by the total number of countries (organizations) in the last decade.

CiteSpace software was used to perform a visualization analysis of key information in the field of global brain science research over the past decade, including important countries, core research institutions, highly productive scholars, highly cited scholars, and highly cited literature. Specifically, the proportion of the top ten highly productive scholars, core research institutions, and highly cited scholars in the United States, the European Union, China, the United Kingdom, and Canada were used as the *X*‐, *Y*‐, and *Z*‐axes, respectively (Figure 9).

The field of global brain science research has clearly been divided into three tiers. The first tier consists only of the United States and the European Union (Germany, France, and Austria), which are globally leading in terms of highly cited scholars and core research institutions, and has a significant lead over the second and third tiers of countries. However, the EU has a lower overall publication output, accounting for only two‐thirds of that of the United States. The second tier consists only of China, which has no advantage in highly cited authors but is on par with the United States in terms of highly published institutions and on par with the European Union in terms of highly published authors. The third tier consists of the major research forces in the global brain science field: the United Kingdom and Canada. At the same time, although China has risen to become the world's second‐largest country in terms of total publication output, only one expert scholar with the highest international influence has emerged (Figure 9). The reality of “following the trend,” “quantity over quality,” and “being scattered and not focused” reminds us that in order to surpass the international leading level and challenge the dominant position of the United States and the European Union in this field, we must deeply analyze the reasons for their absolute and long‐term leading position, especially from the perspective of systematic scientific strategy, action planning, resource allocation, and talent cultivation in research management. We should humbly learn from our experiences and creatively utilize them in combination with our actual national conditions.

## 4 Conclusion

This study, through comprehensive bibliometric analysis, has successfully mapped the global landscape of brain science research, uncovering its trends, hotspots, and collaborative networks. The analysis of 13,590 articles from 1990 to 2023 revealed significant shifts in the field. For instance, China's rise in publication volume, from sixth to second globally post‐2016, signified the growing influence of national initiatives like the China Brain Project. Research hotspots, identified through keyword co‐occurrence and burst detection, evolved from fMRI in 2016 to emerging concepts such as “task analysis” and “deep learning” in 2020–2023.

The implications of these findings are far‐reaching across various professions and research areas. In the medical field, the insights into “Brain Protection” research clusters, which focus on treating brain‐related diseases such as stroke and amyotrophic lateral sclerosis, can guide clinicians in developing more effective treatment strategies. The exploration of brain–computer interfaces and other emerging technologies within the “Brain Creation” cluster holds great potential for rehabilitative medicine, offering new ways to assist patients with neurological disorders in regaining lost functions.

For researchers in artificial intelligence and neuroscience, the identification of research directions such as the development of new learning models inspired by brain neuron activity provides valuable guidance. The understanding of how brain‐like artificial intelligence concepts are emerging in brain science research can fuel further exploration at the intersection of these two fields. This may lead to the creation of more intelligent machines and robots, revolutionizing industries such as robotics and automation.

In conclusion, brain science research is in a dynamic phase of development. While large‐scale government‐led projects worldwide are driving its progress, ethical, legal, and social issues associated with emerging technologies need to be carefully addressed. This study not only provides a valuable resource for understanding the current state of brain science research but also offers practical implications for multiple professions and research areas, guiding future research and development efforts.

## Author Contributions

**Sujuan Zhang**: conceptualization, writing – review and editing, methodology, software, writing – original draft. **Jingyan Gu**: methodology, software, data curation, resources. **Yang Yang**: visualization, data curation, investigation. **Jiangan Li**: writing – review and editing, visualization, supervision. **Lulu Ni**: conceptualization, writing – review and editing, funding acquisition.

## Ethics Statement

All authors have read the journal's position on issues involved in ethical publication and affirm that their report is consistent with those guidelines: “We confirm that we have read the journal's position on issues involved in ethical publication and affirm that this report is consistent with those guidelines.”

## Conflicts of Interest

The authors declare no conflicts of interest.

### Peer Review

The peer review history for this article is available at https://publons.com/publon/10.1002/brb3.70451

## Data Availability

The datasets used and analyzed during the current study are available from the corresponding author upon reasonable request.
